# Survey upon the Reasons of COVID-19 Vaccination Acceptance in Romania

**DOI:** 10.3390/vaccines10101679

**Published:** 2022-10-08

**Authors:** Flavius Cristian Mărcău, Roxana Gheorghițoiu, Iuliana Carmen Bărbăcioru

**Affiliations:** 1 Faculty of Education, Law and Public Administration, “Constantin Brâncuși” University of Târgu Jiu, 210185 Târgu Jiu, Gorj, Romania; 2 Faculty of Medicine, University of Medicine and Pharmacy Craiova, 200349 Craiova, Dolj, Romania; 3 Faculty of Engineering, “Constantin Brâncuși” University of Târgu Jiu, 210185 Târgu Jiu, Gorj, Romania

**Keywords:** vaccination, COVID-19, pandemic, medical education, vaccine acceptance/uptake, social security

## Abstract

Aim: The present study aims to observe the reasons for which the participants have chosen to uptake one of the COVID-19 vaccines approved in Romania. Thus, it will help us to determine whether the reasons are medical in nature, with the respondents’ scope to stay healthy, or if there are other motivations. High rates of vaccine acceptance are essential in the struggle against the COVID-19 pandemic, and trust indicators in other inoculations may be vital for the good management of the vaccination campaign. Methods: The research consisted in applying an online questionnaire in the period January–March 2022 during the fifth wave of COVID-19. The individuals in the target group had to comply with three conditions: they should be inoculated, at least 18 years of age and Romanian residents. The questionnaire was administered to 2297 people and structured to obtain socio-demographic data, determine confidence in mandatory and optional vaccines (rotavirus, hepatitis A, meningococcal vaccine, etc.) and extract the reasons why respondents chose to be vaccinated. Results: The data extracted from the questionnaire reveal a high rate of confidence of participants in the vaccines included in the national vaccination scheme (98.6%) and in the optional vaccines other than anti-COVID-19 (97.2%). Of the respondents, 23.4% had at least one positive test for COVID-19. Although the entire sample is vaccinated against the SARS-CoV-2 virus, the reasons behind the decision to vaccinate are not only medical in nature, thus, 18.3% were motivated by “protecting their own health/protection against the virus”, 17% due to “fear of the disease”, 8.8% for getting back to normal life and ending the pandemic and 8.5% due to government restrictions/vaccination certificate. Conclusions: In our study, we were able to validate the research hypothesis that the reasons for vaccine acceptance are multiple and not only medical (health protection, existing co-morbidities, etc.) and to show that although vaccination has been accepted, some participants believe in conspiracy theories, including those that try to convince people of the harmfulness of the vaccine. In addition, by applying Pearson, Kendall and Spearman correlation tests, we observed that indicators showing high confidence in optional vaccines relate strongly with the decision to vaccinate against COVID-19.

## 1. Introduction

Almost two years have passed since the onset of the COVID-19 pandemic, and its course cannot be predicted due to the multitude of mutations the virus has undergone since its emergence [1,2]. The World Health Organization officially declared the COVID-19 pandemic on 11 March 2020, having previously been considered a public health emergency of international concern (declared on 30 January 2020). The SARS-CoV-2 virus was first identified in the Chinese city of Wuhan (December 2019) but it could not be stopped by traditional quarantine measures in the area, so in a short period, cases of infection appeared in most countries of the world [3].

We are in a time that heralds an acceptance of the virus and a search for ways to live with it [4]. More variants will emerge as a result of the mutations produced and their export from the source state to the rest of the world, making it difficult, if not impossible, to eradicate SARS-CoV-2 [5].

Now, one of the main weapons that has proved able to fight the virus [6] in an effective way is the vaccine against SARS-CoV-2 [6]. Nevertheless, the vaccination rate is not satisfactory in some countries [7,8], due to people’s refusal to receive one of the approved vaccines or booster doses [9]. The reasons behind the rejection or hesitation to be vaccinated against COVID-19 have been addressed in various studies [10,11,12,13,14,15] on the issue in Romania. 

Romania’s vaccination policy has had difficulties in persuading as many people as possible to be immunized. The result of this policy has been a high number of infections, hospitalizations and deaths [15,16]. In 2021, four vaccines were available in Romania to fight SARS-CoV-2: Pfizer (mRNA), Moderna (mRNA), AstraZeneca (non-replicating viral vector) and Johnson & Johnson (non-replicating viral vector). Although the vaccination campaign has been running both in the media and online, it has failed to encourage large numbers of people to get vaccinated. The information presented by officials consisted of urging people to choose vaccination and to obtain information from reliable sources (doctors, officials, etc.). We believe that the low vaccination rate is due to a flawed strategy of promoting real information about vaccination, so that “fake news” information has gained followers [10,11]. The media has played a questionable role to say the least in presenting both pro-vaccination and anti-vaccination views as equally important. Thus, people have become increasingly confused. The Romanian government blamed the poor performance of the vaccination campaign on false news and conspiracy theories circulating in the public space [14].

With this study, we aim to provide qualitative research on the reasons behind the influence and acceptance of vaccination among people in Romania [11]. The adequate number of questionnaires applied (2297) allows us to draw such conclusions and to present the reasons that positively influence the acceptance of COVID-19 vaccine inoculation. Above all, it enables us to determine the confidence of vaccinated people in “fake news”.

The hypothesis of our research is based on the premise that the reasons behind the acceptance of the COVID-19 vaccine are not only medical, but also based on other considerations and, despite being vaccinated, some respondents believe in conspiracy theories that try to convince society of the harmfulness of the vaccine, which raises significant questions.

The purpose of our study is to extract the factors behind why people in Romania agreed to be vaccinated against SARS-CoV-2 and their trust in “fake news”. Thus, the variables we draw from the questionnaire will allow us to answer the following questions, shown in Table 1.

## 2. Materials and Methods

### 2.1. Participants

We have drawn up the present study during the fifth wave of the COVID-19 pandemic, in the period January–March 2022.

In order to carry out our study, we analysed a number of 2297 questionnaires applied within 3 months to people in Romania. To qualify as a target group, a participant had to meet the following conditions:

- Minimum age of 18 at the time of completing the questionnaire;
- Permanent residence in Romania;
- Vaccinated against COVID-19.

The research participants were informed about the nature of the study and their participation was voluntary and unpaid.

### 2.2. Procedure

In view of the proposed research, we developed a questionnaire on the Google Forms platform and distributed it online. Thus, the target social media platform was Facebook (due to the popularity it enjoys in Romania), with the questionnaire being posted in the groups of Romanian cities, groups dedicated to the discussion of vaccination against SARS-CoV-2, and groups created to discuss the specific symptoms of the disease. In addition to Facebook, we used blogs and online media that, during the data collection period, posted the questionnaire for completion by visitors. Further, emails were sent to different databases.

The questionnaire was anonymous, with no respondent identifiers, and could be distributed and completed online by any interested person with no restrictions on electronic de-visits.

### 2.3. Evaluations

The questionnaire consists of two sections:

1. The first encompasses socio-demographic elements, questions concerning the vaccination with the compulsory/optional inoculations, confidence in the vaccines and the reasons influencing the COVID-19 immunization;
2. The second section comprises questions intended to enable our understanding of the respondents’ degree of confidence in the “fake news” allegations regarding the pandemic and the vaccination against COVID-19.

### 2.4. Statistical Analysis of the Data

Data analysis and processing were performed using Excel, which is part of the Microsoft Office Professional Plus 2019 package, and IBM SPSS Statistics 26, which was installed on a computer with a Windows 11 Professional operating system.

The processed data obtained from the questionnaire were analysed both statistically and analytically. The variables used for the analysis are:

(a). Age range;
(b). Level of education;
(c). Residence environment;
(d). Acceptance of compulsory vaccines included in the national vaccination scheme and optional vaccines other than anti-COVID-19 (rotavirus, anti-hepatitis A, meningococcal vaccine, etc.);
(e). Reasons for accepting the COVID-19 vaccine;
(f). The participants’ degree of trust in the “fake news”;

The extracted dataset was statistically analysed in order to obtain the percentages, frequency distribution, medians and standard deviation, and in order to establish correlations between different variables we used Pearson, Kendall and Spearman statistical tests.

## 3. Results

The study was based on 2297 valid questionnaires. The socio-demographic data of the respondents are shown in Table 2.

The participants’ confidence in the vaccines included in Romania’s national vaccination scheme (98.6%) and the percentage of vaccinated (99.4%) by age category are shown in Table 3.

Elective vaccines, such as flu vaccines, hepatitis vaccines, HPV vaccines, etc., received significantly less confidence from participants (97.2%), according to Table 4.

The participants who officially passed COVID-19 (23.4%) with at least one positive test, by age category, are shown in Table 5.

The opinions of the participants are divided when the topic of mandatory vaccination against COVID-19 is introduced, with 68.3% of them considering it appropriate, while 31.6% are against, as shown in Table 6.

The medical, psychological, civic, etc., arguments underlying the decision to vaccinate against COVID-19 are given in Table 7.

Although the entire sample surveyed had been vaccinated with one of the vaccines approved in Romania, some of the participants trust information classified as “fake news”, as shown in Table 8.

## 4. Discussion

The participants place high confidence in vaccines included in the national vaccination scheme (98.6%) and in optional vaccines (97.2%) other than anti-COVID-19 (rotavirus, hepatitis A, meningococcal vaccine, etc.). The lowest confidence rate is found in the 18–20 age group (94.7%), followed by the 21–25 age group (95.3%). Studies [11] show that confidence in the mandatory vaccines administered during childhood is not a constant factor in people’s decision to vaccinate against COVID-19 [17,18,19,20], since the decision did not belong to them at that time (being minors, parents/legal guardians decided for them). Moreover, according to Mărcău et al. [10,11], people who chose not to vaccinate place sufficiently high confidence in the optional vaccines, but do not place confidence in the SARS-CoV-2 vaccines [10,11]. The minimum confidence rate for the optional vaccines (Table 4) is represented by the age groups 21–25 years (91.8%) and 66+ (93.3%).

In our research, given that the entire sample is vaccinated against SARS-CoV-2, we find an extremely strong correlation between the variable “confidence in optional vaccines” and the participants’ decision to vaccinate against COVID-19, the results of which are shown in Table 9.

Hence, according to the above-mentioned data, we understand that the participants who choose to trust the optional vaccines that are available on the Romanian market and administered on demand chose to be inoculated by one of the COVID-19 vaccines approved in Romania, whereas the correlation tests applied demonstrate a very strong link between the two variables, the correlation coefficient being 1 and sig. 0.000.

The possibility of mandatory COVID-19 vaccination in Romania has provoked different reactions in the population. Such reactions, for or against, were also found among the participants surveyed, with 68.3% of them considering mandatory vaccination against the SARS-CoV-2 virus beneficial. The lowest rate of support for such a measure was observed (Table 5) in the age groups 21–25 years (55.8%) and 18–20 years (58.2%).

Although the entire sample was vaccinated with one of the COVID-19 vaccines approved in Romania, only 23.4% of the participants had officially experienced the COVID-19 disease (Table 5), having had at least one positive test by the time the questionnaire was completed.

Moreover, the arguments behind the decision to vaccinate (Table 7) are not only of a medical nature. In addition to the participants’ desire to remain healthy and cautious against COVID-19 (18.3%), we find “fear of getting sick” (17%) or “the desire to protect others” (11.6%) to be strong considerations in vaccination decisions. However, we also find other reasons, unrelated to concerns about individual health, which led the participants to vaccinate. Thus, “desire to end the pandemic” (8.8%) in order to return to normal life and “government restrictions/need for a vaccination certificate” (8.5%) are reasons expressing participants’ desire to move freely without encountering the restrictions imposed on unvaccinated people, not necessarily confidence in the usefulness of the COVID-19 vaccine.

The level of trust that participants show in “fake news” information, categorized as conspiracy theories against the vaccine, is significantly lower than for people who did not choose to vaccinate against COVID-19 [10,11]. In the case of the sample presented in our research, we find extremely low trust in such information (Table 8). Of the participants, 1.8% believe that the COVID-19 pandemic is not real (A1), and 7% believe that there is a secret global organization that wants to control the world (A2). Likewise, 8.8% of respondents believe that vaccination against SARS-CoV-2 is not aimed at eradicating SARS-CoV-2 (A9), and 5.9% believe that vaccination is aimed at enriching vaccine manufacturers (A11). Regarding the statement “New messenger RNA-based vaccines produce dangerous genetic changes”, 3.26% of the participants trust it (A10). A total of 2.3% of the respondents believe that COVID-19 vaccines were made to help reduce the Earth’s population (A3). Although the percentage of people who believe in such conspiracy theories is small (in our sample), they chose to vaccinate against SARS-CoV-2, most likely due to government restrictions, to obtain their vaccination certificate or to be able to perform their job duties (due to constraints).

## 5. Research Limitations

Although our study has many strengths, there are also some limitations of the research. A first limitation is that the percentage of people in rural areas is lower than in urban areas. We believe that a higher number would have increased the percentages of trust given to fake news claims [7,21,22,23,24]. A second limitation is that the research was conducted during wave five, and Romania reported the highest rates of increase in the number of infected since the beginning of the pandemic, so some participants chose to vaccinate due to fear generated by official reports or media reports. Although the research is qualitative, based on the data obtained from the questionnaire, the third limitation is closely related to the fact that the study was conducted online, with the possibility of a subjective self-selection of participants [25]. Moreover, only those participants who had access to the Internet were able to respond to the questionnaire; many people over 60 use the Internet very little, if at all, let alone social media platforms. Ball HL is also of the opinion that respondents may share the survey with friends and colleagues with similar interests or perspectives, which may lead to over-representation of a particular point of view [26]. On the other hand, the online survey may be tainted by the possibility of deceptive practices, as participants may intentionally provide erroneous responses due to strong feelings they have and wish to see represented [26].

## 6. Conclusions

Through the proposed study, we were able to validate our research hypothesis, showing that the reasons behind the decision to vaccinate against COVID-19 are not only medical in nature, and that some of the respondents, although they agreed to be vaccinated, believe in conspiracy theories. The participants who chose to be inoculated with one of the vaccines approved in Romania did so not only because they wanted to protect their own health, the health of others around them or due to the existence of co-morbidities, but also because of the restrictive measures imposed by the government on non-vaccinated people (8.5%), which included a ban on entering certain commercial premises, the right to travel, freedom of movement after certain hours, etc. Therefore, we can conclude that in addition to medical and self-preservation reasons, there are also other compelling or civic reasons (Table 7), which were also noted when determining participants’ level of trust in “fake news” information. Despite choosing to vaccinate, some participants believe conspiracy information (3.2% believe that messenger RNA causes dangerous genetic changes) or that vaccines were designed to reduce the world’s population (2.3%).

Furthermore, in the case of our study, we observed a strong correlation between the variables “confidence in optional vaccines” and the participants’ decision to vaccinate against COVID-19; however, this cannot be considered unanimously valid given that Mărcău et al. [10] demonstrated that a high percentage of confidence in optional vaccines does not lead participants to accept the COVID-19 vaccine.

The vaccination campaign has encountered difficulties in effectively transmitting information to citizens, so Romania has one of the lowest vaccination rates in Europe. The lack of a minimum medical culture among citizens is to blame for the rate of reliance some participants place on conspiracy theories and for the high number of people refusing/hesitating to be vaccinated against SARS-CoV-2.

A perspective for future research is the need to conduct studies on the degree of Romanian citizens’ medical literacy, so we can be better understand the beliefs of the population and their decisions to accept/reject certain treatments/vaccines, etc., in the case of potential future pandemic scenarios. We are of the opinion that such studies are necessary for the correct implementation of an effective information campaign and, above all, to avoid increasing public confidence in false information.

## Figures and Tables

**Table 1:** Necessary data and questions.

**Necessary Data**	**Questions**
Participants’ trust in the optional vaccines other than COVID-19 (rotavirus, hepatitis a, meningococcal vaccine, etc.)	Is there any relation between the uptake of the optional vaccines and acceptance of the anti-COVID-19 one?
Factors influencing vaccine acceptance	Are the reasons given by participants only of a medical nature (protection of their own health)?
Participants’ confidence in “fake news allegations”	Do the respondents who chose to get vaccinated believe in conspiracy theories about the COVID-19 pandemic?

**Table 2:** Socio-demographic data.

Age	Sex					Environment of Residence		Educational Level				
	Female		Male		N.A.	Urban %	Rural %	Second. Educ. %	High School %	Faculty %	Masters %	PhD. %
	N	%	N	%	%							
18–20	83	3.6	31	1.3	0.04	3.5	1.4	0.09	1.5	3.4	0	0
21–25	177	7.7	79	3.4	0	8.4	2.7	0	1.4	7.4	2.1	0.04
26–30	83	3.6	106	4.6	0	6.8	1.3	0.04	0.8	3.7	3.4	0.1
31–35	155	6.7	227	9.8	0	14.1	2.4	0.04	1.6	7.5	7.1	0.3
36–40	160	6.9	298	12.9	0	17.4	2.5	0.1	2.3	10.4	6.2	0.7
41–45	153	6.6	283	12.3	0.09	16.3	2.7	0.1	2.6	10.6	5	0.6
46–50	101	4.4	135	5.8	0	8.4	1.8	0.04	1.7	5	2.3	1
51–55	69	3	63	2.7	0.04	5.3	0.4	0.09	1.2	2.4	1.6	0.3
56–60	32	1.3	18	0.7	0	2	0.1	0.04	0.4	1	0.4	0.2
61–65	10	0.4	15	0.6	0	0.9	0.1	0	0.2	0.4	0.2	0.1
66+	9	0.3	6	0.2	0	0.6	0.04	0	0.2	0.2	0.04	0.1

**Table 3:** Confidence and vaccination rate of participants in/with vaccines included in the national vaccination scheme.

Age Range	Do You Trust the Mandatory Childhood Vaccines?	Have You Been Vaccinated with the Mandatory Childhood Inoculations?
	Yes %	Yes %
18–20	94.7	98.2
21–25	95.3	98.8
26–30	98.9	99.4
31–35	99.4	99.7
36–40	99.1	99.5
41–45	99.5	99.7
46–50	99.1	100
51–55	100	99.2
56–60	98	100
61–65	100	96
66+	100	100
**Descriptive statistics**		
Mean	0.98576	0.99171
Standard Error	0.005557	0.003567
Median	0.991525	0.995633
Standard Deviation	0.018431	0.01183
Sample Variance	0.00034	0.00014
Kurtosis	1.150305	5.520393
Skewness	−1.53874	−2.25037
Confidence Level (95.0%)	0.012382	0.007948

**Table 4:** Participants’ trust in the optional/elective vaccines.

Age Range	Do You Trust in the Elective Vaccines (Flu Vaccines, Hepatitis Vaccines, HPV Vaccines, Etc.)?
	Yes %
18–20	96.5
21–25	91.8
26–30	97.3
31–35	98.1
36–40	98.2
41–45	98.6
46–50	97.8
51–55	96.9
56–60	96
61–65	100
66+	93.3
**Descriptive statistics**	
Mean	0.968119
Standard Error	0.007189
Median	0.973545
Standard Deviation	0.023843
Sample Variance	0.000569
Kurtosis	0.954189
Skewness	−1.11355
Confidence Level (95.0%)	0.016018

**Table 5:** SARS-CoV-2 infection frequency of participants.

Age Range	Have You Officially Passed through SARS-CoV-2 (COVID-19)?
	Yes %
18–20	19.1
21–25	25.3
26–30	22.2
31–35	21.9
36–40	26.6
41–45	22.6
46–50	25.8
51–55	21.8
56–60	14
61–65	24
66+	13.3
**Descriptive statistics**	
Mean	0.215417
Standard Error	0.013392
Median	0.222222
Standard Deviation	0.044418
Sample Variance	0.001973
Kurtosis	0.11191
Skewness	−0.99076
Confidence Level (95.0%)	0.02984

**Table 6:** Mandatory vaccination uptake rate.

Age Range	Do You Think That COVID-19 Vaccination Should Be Mandatory in Romania?
	Yes %
18–20	58.2
21–25	55.8
26–30	68.7
31–35	68.5
36–40	69.4
41–45	70.7
46–50	70.3
51–55	77.4
56–60	80
61–65	84
66+	66.6
**Descriptive statistics**	
Mean	0.700134
Standard Error	0.025313
Median	0.694323
Standard Deviation	0.083952
Sample Variance	0.007048
Kurtosis	−0.08444
Skewness	−0.07406
Confidence Level (95.0%)	0.0564

**Table 7:** Participants’ arguments underlying the decision to vaccinate against COVID-19.

Arguments	N	%
Health/protection	422	18.3
Fear of disease	391	17
Protect those around them (friends, family, colleagues, etc.)	267	11.6
End of pandemic	203	8.8
Governmental restrictions/green certificate	196	8.5
Confidence in medicine and science	165	7.1
Trust in vaccines	155	6.7
Civic duty	104	4.5
Passing through the disease	51	2.2
High rate of cases	46	2
Infected friends/family	30	1.3
Existing co-morbidities	29	1.2
Deaths in the family	22	0.9
Other reasons	216	9.4

**Table 8:** Participants’ trust in “fake news” [10,11].

Fake News Allegations		Disagree (1–2)		Unsure (3)		Agree (4–5)	
		N	%	N	%	N	%
A1	The pandemic of COVID-19 is real	42	1.8	79	3.4	2176	94.7
A2	There’s a global conspiracy that wants to control the world	1948	84.8	188	8.1	161	7
A3	COVID-19 vaccines are made to reduce Earth’s population	2171	94.5	71	3	55	2.3
A4	Doctors are paid to inoculate a vaccine that would help reduce the Earth’s population	2214	96.3	47	2	36	1.5
A5	People who chose to take the COVID-19 vaccine will die in the next few years due to inoculated substances	2212	96.2	52	2.2	33	1.4
A6	The COVID-19 vaccine is intended to implant a CIP in the body	2261	98.4	18	0.7	18	0.7
A7	Vaccination is intended to reduce the number of elderly people	2220	96.6	36	1.5	41	1.7
A8	There’s a global occult that wants to reduce the Earth’s population	2122	92.3	102	4.4	73	3.1
A9	Vaccination aims to eradicate COVID-19	203	8.8	133	5.7	1961	85.3
A10	New messenger RNA-based vaccines produce dangerous genetic changes	2082	90.6	140	6	75	3.2
A11	Global vaccination aims to enrich vaccine manufacturers	1929	83.9	231	10	137	5.9

**Table 9:** Correlation between the variable “confidence in optional vaccines” and participants’ decision to vaccinate against COVID-19.

** 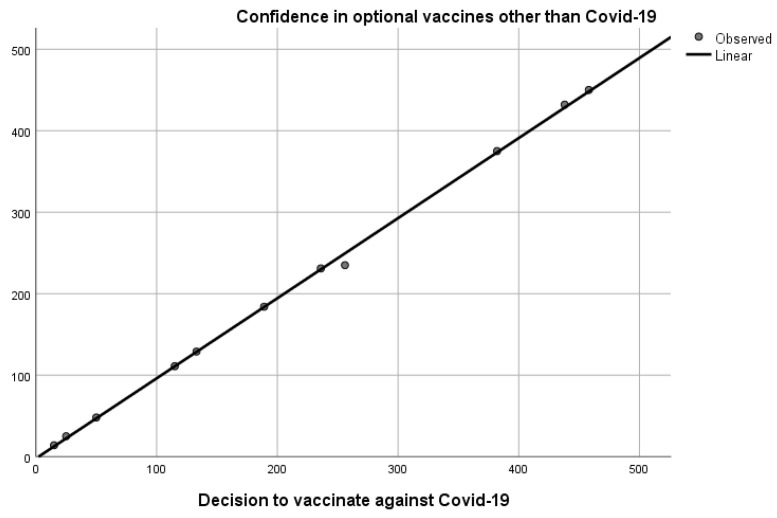 **		
Kendall	Correlation coefficient	1.000
	Sig. (2-tailed)	0.000
Spearman	Correlation coefficient	1.000
	Sig. (2-tailed)	0.000
Pearson	Correlation coefficient	1.000
	Sig. (2-tailed)	0.000

## Data Availability

Data can be requested from the corresponding author.
